# Innovation for healthy ageing: a call for papers

**DOI:** 10.2471/BLT.16.176743

**Published:** 2016-06-01

**Authors:** Islene Araujo de Carvalho, Isabella Aboderin, Eri Arikawa-Hirasawa, Matteo Cesari, Yoshiaki Furukawa, Luis Miguel Gutierrez Robledo, John E Morley, Anne Margriet Pot, Jean-Yves Reginster, Greg Shaw, Naoko Tomita, John R Beard

**Affiliations:** a World Health Organization, avenue Appia 20, 1211 Geneva 27, Switzerland.; b Ageing and Development Programme, African Population and Health Research Center, Nairobi, Kenya.; c Graduate School of Medicine, Jutendo University, Tokyo, Japan.; d Gérontopôle, Centre Hospitalier Universitaire de Toulouse, Toulouse, France.; e Instituto Nacional de Geriatría de México, Mexico City, Mexico.; f Division of Geriatrics and Endocrinology, St Louis University, St Louis, United States of America.; g Department of Public Health, Epidemiology and Health Economics, University of Liège, Liège, Belgium.; h International Federation on Ageing, Toronto, Canada.; i Department of International Health and Collaboration, National Institute of Public Health, Saitama, Japan.

In most regions, over the past 50 years, socioeconomic development has been accompanied by large falls in fertility and equally dramatic increases in life expectancy.^1^ This phenomenon has led to rapidly ageing populations around the world. The fastest change is occurring in low- and-middle-income countries. Even in sub-Saharan Africa, which has the world’s youngest population structure, the number of people older than 60 years is expected to increase by over threefold, from 46 million in 2015 to 147 million in 2050.^2^

Increasing life expectancy presents many opportunities to individuals as well as the communities they live in. Older people contribute to society in many ways, for example, through participation in the workforce, the taxes they pay, the direct economic support they can give to younger family members, or through the sharing of their experience.^3^ Even in high-income countries that have comprehensive social protection platforms, the economic value of these contributions outweigh the direct costs of pensions, health care and other services that governments provide.^3^

However, the extent of these opportunities and contributions will depend heavily on the health of these older populations. In rich countries, it is often assumed that older people live these later years of life in good health. Unfortunately, while there is some evidence that cognitive declines may be occurring at later ages than seen in the past, there is very little evidence that older people today are enjoying better physical capacity than their parents did at the same age.^4^^,^^5^ In low- and middle- income countries, older people experience even higher rates of ill-health and impaired function.^6^ Yet this does not have to be the case. Most poor health in older age is the consequence of chronic diseases, many of which can be prevented, or, if detected early, can be effectively controlled. Even in cases where older people experience declines in capacity, supportive environments can ensure that they continue to live their lives with meaning and dignity.

Increased longevity is one of the great achievements of the 20th century. Ensuring the added years can be enjoyed in good health will be one of the biggest public health challenges of the first half of the 21st century. Addressing this challenge will require changing perceptions and assumptions about health in older age. Changes are also needed in the way that health systems are designed and the ways in which care is delivered and measured.

Several global initiatives are shaping discussions on how these challenges might be addressed. The first ever *World report on ageing and health*^1^ was released in 2015 and the *Global Strategy and Implementation Plan on Ageing and Health* will be considered at the 2016 World Health Assembly. The report presented a conceptual framework for healthy ageing built around the functional ability of older people, rather than the absence of disease. It highlighted knowledge gaps as a major barrier to global action. It also emphasized that any action to address healthy ageing requires sound evidence stemming from careful evaluation of cost-effective interventions. 

Evidence on how to ensure healthy ageing, particularly in people living in low- and middle-income countries, is scarce. This is partly because the transition to an ageing population in these countries has been relatively recent and more rapid than in high income countries. The limited research that has been conducted on the effectiveness of relevant interventions has been done mostly in high-income countries.

The *Bulletin of the World Health Organization* will publish a theme issue on actions and approaches to support the development of resilient health and long-term care systems for ageing populations. This theme issue will include original research, examine available knowledge and share evidence on best practices around healthy ageing. It will include papers that will highlight the interconnectedness of health and social issues in maintaining healthy ageing and how the combination of appropriate health and social strategies can promote functional ability and lead to a happier and healthier older population.

We welcome papers for all sections of the *Bulletin*, around the following themes: (i) use of technologies and innovations to improve functional ability and promote healthy ageing; (ii) effectiveness of public health and clinical interventions to prevent and reverse declines in physical and mental capacities; (iii) community-based public health interventions to support caregivers of older people; (iv) determinants of different trajectories of function; (v) long-term care systems in low- and middle- income countries; (vi) elements of effective integrated care service delivery models; (vii) management of geriatric conditions, such as frailty, sarcopenia, urinary incontinence, and dementia.

The deadline for submission is 1 November 2016. Manuscripts should be submitted in accordance with the *Bulletin’s Guidelines for contributors* (http://submit.bwho.org), and the cover letter should mention this call for papers. 
